# *cis*-Chloridobis(4,4′-dimethyl-2,2′-bipyridine-κ^2^ *N*,*N*′)oxidovanadium(IV) chloride ethanol monosolvate monohydrate

**DOI:** 10.1107/S1600536812040251

**Published:** 2012-09-29

**Authors:** Sadif A. Shirvan, Sara Haydari Dezfuli, Elyas Golabi, Mohammad Amin Gholamzadeh

**Affiliations:** a Department of Chemistry, Islamic Azad University, Omidieh Branch, Omidieh, Iran; b Department of Petroleum Engineering, Islamic Azad University, Omidieh Branch, Omidieh, Iran

## Abstract

In the title compound, [VClO(C_12_H_12_N_2_)_2_]Cl·C_2_H_5_OH·H_2_O, the V^IV^ atom is six-coordinated in a distorted octa­hedral geometry by four N atoms from two 4,4′-dimethyl-2,2′-bipyridine ligands, one O atom and one Cl atom. In the crystal, O—H⋯Cl, C—H⋯O and C—H⋯Cl hydrogen bonds and π–π contacts between the pyridine rings [centroid–centroid distances = 3.7236 (17) and 3.6026 (19) Å] stabilize the structure. Intra­molecular C—H⋯O and C—H⋯Cl hydrogen bonds are also present.

## Related literature

For related structures, see: Ahmadi *et al.* (2008 ▶); Alizadeh *et al.* (2010 ▶); Amani *et al.* (2009 ▶); Hojjat Kashani *et al.* (2008 ▶); Kalateh *et al.* (2008 ▶, 2010 ▶); Shirvan & Haydari Dezfuli (2011 ▶, 2012*a* ▶,*b* ▶); Triantafillou *et al.* (2004 ▶); Yousefi *et al.* (2008 ▶).
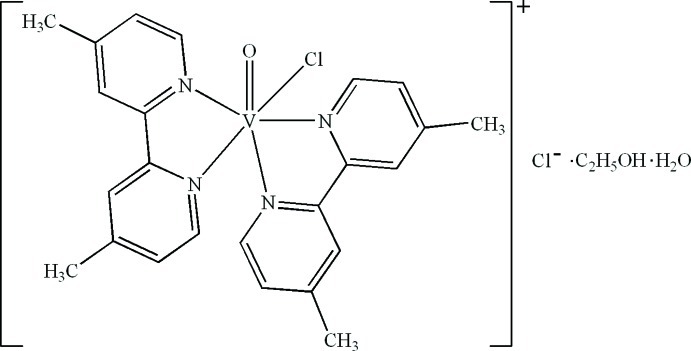


## Experimental

### 

#### Crystal data

[VClO(C_12_H_12_N_2_)_2_]Cl·C_2_H_6_O·H_2_O
*M* *_r_* = 570.40
Triclinic, 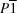

*a* = 9.6955 (11) Å
*b* = 10.9905 (12) Å
*c* = 14.5739 (17) Å
α = 68.711 (2)°
β = 72.401 (2)°
γ = 81.381 (2)°
*V* = 1377.9 (3) Å^3^
*Z* = 2
Mo *K*α radiation
μ = 0.59 mm^−1^
*T* = 120 K
0.32 × 0.14 × 0.09 mm

#### Data collection

Bruker SMART 1000 CCD diffractometer
Absorption correction: multi-scan (*SADABS*; Sheldrick, 1996 ▶) *T*_min_ = 0.926, *T*_max_ = 0.954
12257 measured reflections
5363 independent reflections
4398 reflections with *I* > 2σ(*I*)
*R*_int_ = 0.026

#### Refinement

*R*[*F* ^2^ > 2σ(*F* ^2^)] = 0.062
*wR*(*F* ^2^) = 0.144
*S* = 1.00
5363 reflections
330 parameters
H-atom parameters constrained
Δρ_max_ = 2.09 e Å^−3^
Δρ_min_ = −0.89 e Å^−3^

### 

Data collection: *SMART* (Bruker, 2007 ▶); cell refinement: *SAINT* (Bruker, 2007 ▶); data reduction: *SAINT*; program(s) used to solve structure: *SHELXS97* (Sheldrick, 2008 ▶); program(s) used to refine structure: *SHELXL97* (Sheldrick, 2008 ▶); molecular graphics: *SHELXTL* (Sheldrick, 2008 ▶) and *Mercury* (Macrae *et al.*, 2006 ▶); software used to prepare material for publication: *SHELXTL*.

## Supplementary Material

Crystal structure: contains datablock(s) I, global. DOI: 10.1107/S1600536812040251/hy2588sup1.cif

Structure factors: contains datablock(s) I. DOI: 10.1107/S1600536812040251/hy2588Isup2.hkl

Additional supplementary materials:  crystallographic information; 3D view; checkCIF report

## Figures and Tables

**Table 1:** Hydrogen-bond geometry (Å, °)

*D*—H⋯*A*	*D*—H	H⋯*A*	*D*⋯*A*	*D*—H⋯*A*
O1*S*—H1*S*⋯Cl2	0.93	2.24	3.128 (3)	159
O1*W*—H1*W*⋯Cl2^i^	0.84	2.45	3.221 (3)	152
O1*W*—H2*W*⋯Cl2^ii^	0.92	2.37	3.284 (3)	171
C1—H1*B*⋯O1	0.95	2.51	2.999 (4)	112
C2—H2*A*⋯O1*S* ^iii^	0.95	2.41	3.301 (4)	155
C4—H4*A*⋯Cl1^iv^	0.95	2.76	3.670 (4)	160
C7—H7*A*⋯Cl1^iv^	0.95	2.77	3.626 (3)	151
C9—H9*A*⋯Cl2^ii^	0.95	2.63	3.561 (4)	165
C13—H13*A*⋯O1*S* ^iv^	0.95	2.59	3.284 (4)	130
C14—H14*A*⋯Cl2^iv^	0.95	2.62	3.558 (3)	170
C19—H19*A*⋯O1^v^	0.95	2.52	3.229 (4)	132
C21—H21*A*⋯O1*W* ^vi^	0.95	2.45	3.315 (5)	151
C22—H22*A*⋯Cl1	0.95	2.64	3.262 (3)	123

Symmetry codes: (i) 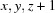; (ii) 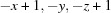; (iii) 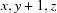; (iv) 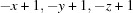; (v) 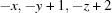; (vi) 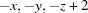.

**Fig. 1.**
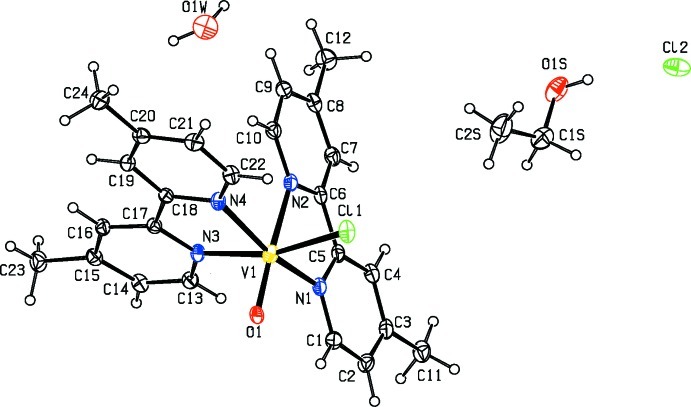
The molecular structure of the title compound. Displacement ellipsoids are drawn at the 30% probability level.

**Fig. 2.**
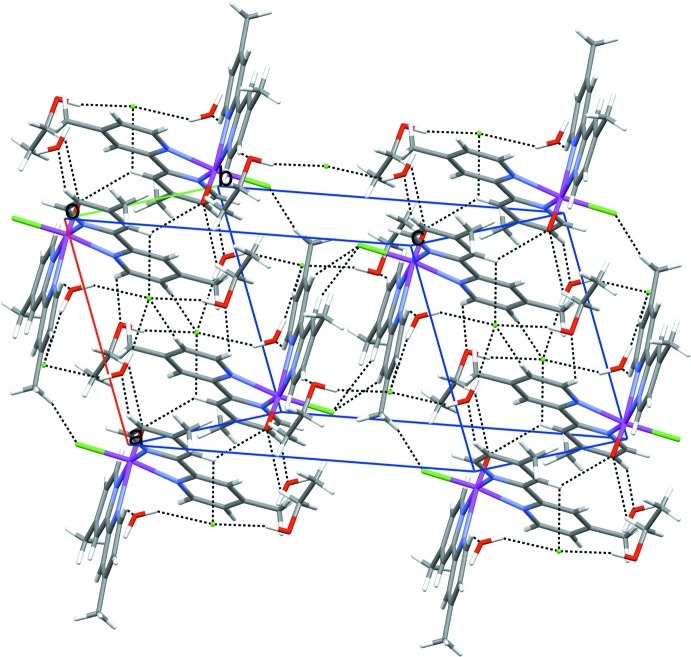
Crystal packing of the title compound. Hydrogen bonds are shown as dashed lines.

## supplementary crystallographic information

### Comment

Recently, we reported the synthesis and crystal structures of [In(4,4'-dmbipy)Cl_3_(MeOH)].MeOH, (II) (Shirvan & Haydari Dezfuli, 2012*b*), and [CdBr_2_(4,4'-dmbipy)(DMSO)], (III) (Shirvan & Haydari Dezfuli, 2012*a*) (4,4'-dmbipy = 4,4'-dimethyl-2,2'-bipyridine, DMSO = dimethyl sulfoxide). 4,4'-Dmbipy is a good bidentate ligand, and numerous complexes with 4,4'-dmbipy have been prepared, such as that of [Hg(4,4'-dmbipy)I_2_], (IV) (Yousefi *et al.*, 2008), [Hg(4,4'-dmbipy)Br_2_], (V) (Kalateh *et al.*, 2008), [Fe(4,4'-dmbipy)Cl_3_(DMSO)], (VI) (Amani *et al.*, 2009), [Pt(4,4'-dmbipy)Cl_4_], (VII) (Hojjat Kashani *et al.*, 2008), [Cd(4,4'-dmbipy)I_2_(DMSO)], (VIII) (Kalateh *et al.*, 2010), [Zn(4,4'-dmbipy)Br_2_], (IX) (Alizadeh *et al.*, 2010), [Zn(4,4'-dmbipy)(H_2_O)(NO_3_)_2_], (X) (Shirvan & Haydari Dezfuli, 2011) and [In(4,4'-dmbpy)Cl_3_(DMSO)], (XI) (Ahmadi *et al.*, 2008). We report herein the synthesis and crystal structure of the title compound, (I).

In the title compound (Fig. 1), the V^IV^ atom is six-coordinated in a distorted octahedral geometry by four N atoms from two 4,4'-dmbipy ligands, one O atom and one Cl atom. There are also one ethanol and one water solvent molecules in the asymmetric unit. The V—Cl, V—N and V—O bond lengths and angles are in good agreement with the corresponding values in [VOCl(dtbipy)Cl]Cl.CH_2_Cl_2_ (Triantafillou *et al.*, 2004) (dtbipy = 4,4'-di-tert-butyl-2,2'-bipyridine).

In the crystal, intermolecular O—H···Cl, C—H···O and C—H···Cl hydrogen bonds, intramolecular C—H···O and C—H···Cl hydrogen bonds (Table 1) and π–π contacts between the pyridine rings (Fig. 2), *Cg*3···*Cg*4^i^ and *Cg*5···*Cg*6^ii^, with centroid–centroid distances of 3.7236 (17) and 3.6026 (19) Å [symmetry codes: (i) 1-x, 1-y, 1-z; (ii) -x, 1-y, 2-z. *Cg*3, *Cg*4, *Cg*5 and *Cg*6 are the centroids of the N1/C1–C5, N2/C6–C10, N3/C13–C17 and N4/C18–C22 rings, respectively], stabilize the structure.

### Experimental

For the preparation of the title compound, a solution of 4,4'-dimethyl-2,2'-bipyridine (0.29 g, 1.60 mmol) in ethanol (20 ml) was added to a solution of VCl_3_ (0.13 g, 0.80 mmol) in water (4 ml) and the resulting violet solution was stirred at 323 K for 40 min. Then, it was left to evaporate slowly at room temperature. After six days, green prismatic crystals of the title compound were isolated (yield: 0.32 g, 74.0%).

### Refinement

H atoms bonded to C atoms were positioned geometrically and refined as riding atoms, with C—H = 0.95 (aromatic), 0.99 (CH_2_) and 0.98 (CH_3_) Å and with *U*_iso_(H) = 1.2(1.5 for methyl)*U*_eq_(C). H atoms of hydroxyl group and water molecules were located in a difference Fourier map and refined as riding atoms, with *U*_iso_(H) = 1.2*U*_eq_(O). There is a high residual peak of 2.09 e Å^-3^ near V1 atom due to the absorption effects that could not be correctly account for. The highest residual electron density was found at 0.84 Å from V1 atom and the deepest hole at 0.65 Å from V1 atom.

### Crystal data

**Table:**

[VClO(C_12_H_12_N_2_)_2_]Cl·C_2_H_6_O·H_2_O	*Z* = 2
*M**_r_* = 570.40	*F*(000) = 594
Triclinic, *P*1	*D*_x_ = 1.375 Mg m^−^^3^
Hall symbol: -P 1	Mo *K*α radiation, λ = 0.71073 Å
*a* = 9.6955 (11) Å	Cell parameters from 600 reflections
*b* = 10.9905 (12) Å	θ = 3.0–26.0°
*c* = 14.5739 (17) Å	µ = 0.59 mm^−^^1^
α = 68.711 (2)°	*T* = 120 K
β = 72.401 (2)°	Prism, green
γ = 81.381 (2)°	0.32 × 0.14 × 0.09 mm
*V* = 1377.9 (3) Å^3^	

### Data collection

**Table:**

Bruker SMART 1000 CCD diffractometer	5363 independent reflections
Radiation source: fine-focus sealed tube	4398 reflections with *I* > 2σ(*I*)
Graphite monochromator	*R*_int_ = 0.026
φ and ω scans	θ_max_ = 26.0°, θ_min_ = 2.0°
Absorption correction: multi-scan (*SADABS*; Sheldrick, 1996)	*h* = −11→11
*T*_min_ = 0.926, *T*_max_ = 0.954	*k* = −13→13
12257 measured reflections	*l* = −17→17

### Refinement

**Table:**

Refinement on *F*^2^	Primary atom site location: structure-invariant direct methods
Least-squares matrix: full	Secondary atom site location: difference Fourier map
*R*[*F*^2^ > 2σ(*F*^2^)] = 0.062	Hydrogen site location: inferred from neighbouring sites
*wR*(*F*^2^) = 0.144	H-atom parameters constrained
*S* = 1.00	*w* = 1/[σ^2^(*F*_o_^2^) + (0.048*P*)^2^ + 3.916*P*] where *P* = (*F*_o_^2^ + 2*F*_c_^2^)/3
5363 reflections	(Δ/σ)_max_ < 0.001
330 parameters	Δρ_max_ = 2.09 e Å^−^^3^
0 restraints	Δρ_min_ = −0.89 e Å^−^^3^

### Special details

**Table:**

Geometry. All e.s.d.'s (except the e.s.d. in the dihedral angle between two l.s. planes) are estimated using the full covariance matrix. The cell e.s.d.'s are taken into account individually in the estimation of e.s.d.'s in distances, angles and torsion angles; correlations between e.s.d.'s in cell parameters are only used when they are defined by crystal symmetry. An approximate (isotropic) treatment of cell e.s.d.'s is used for estimating e.s.d.'s involving l.s. planes.
Refinement. Refinement of *F*^2^ against ALL reflections. The weighted *R*-factor *wR* and goodness of fit *S* are based on *F*^2^, conventional *R*-factors *R* are based on *F*, with *F* set to zero for negative *F*^2^. The threshold expression of *F*^2^ > σ(*F*^2^) is used only for calculating *R*-factors(gt) *etc*. and is not relevant to the choice of reflections for refinement. *R*-factors based on *F*^2^ are statistically about twice as large as those based on *F*, and *R*- factors based on ALL data will be even larger.

### Fractional atomic coordinates and isotropic or equivalent isotropic displacement parameters (Å^2^)

**Table:**

	*x*	*y*	*z*	*U*_iso_*/*U*_eq_	
V1	0.17871 (6)	0.53590 (5)	0.73759 (4)	0.02822 (16)	
Cl1	0.10808 (9)	0.45573 (9)	0.63211 (6)	0.0368 (2)	
O1	0.0514 (2)	0.6517 (2)	0.74454 (15)	0.0278 (5)	
N1	0.3293 (3)	0.6612 (3)	0.61322 (19)	0.0269 (6)	
N2	0.3918 (3)	0.4168 (2)	0.71332 (19)	0.0259 (6)	
N4	0.0941 (3)	0.3772 (3)	0.86974 (19)	0.0252 (5)	
N3	0.2635 (3)	0.5586 (3)	0.84871 (18)	0.0240 (5)	
C1	0.2904 (4)	0.7853 (3)	0.5636 (2)	0.0312 (7)	
H1B	0.1937	0.8162	0.5863	0.037*	
C2	0.3840 (4)	0.8699 (3)	0.4815 (2)	0.0327 (7)	
H2A	0.3525	0.9572	0.4496	0.039*	
C3	0.5253 (4)	0.8251 (3)	0.4462 (2)	0.0320 (7)	
C4	0.5655 (4)	0.6968 (3)	0.4970 (2)	0.0302 (7)	
H4A	0.6610	0.6634	0.4745	0.036*	
C5	0.4672 (4)	0.6175 (3)	0.5801 (2)	0.0263 (7)	
C6	0.5039 (3)	0.4814 (3)	0.6387 (2)	0.0260 (6)	
C7	0.6429 (4)	0.4240 (3)	0.6201 (2)	0.0301 (7)	
H7A	0.7195	0.4724	0.5674	0.036*	
C8	0.6698 (4)	0.2963 (3)	0.6784 (2)	0.0308 (7)	
C9	0.5540 (4)	0.2300 (3)	0.7545 (2)	0.0304 (7)	
H9A	0.5676	0.1422	0.7961	0.036*	
C10	0.4187 (4)	0.2928 (3)	0.7691 (2)	0.0279 (7)	
H10A	0.3406	0.2458	0.8213	0.033*	
C11	0.6322 (4)	0.9131 (4)	0.3567 (3)	0.0401 (9)	
H11A	0.5933	0.9458	0.2970	0.060*	
H11B	0.7239	0.8636	0.3412	0.060*	
H11C	0.6488	0.9870	0.3740	0.060*	
C12	0.8190 (4)	0.2308 (4)	0.6607 (3)	0.0419 (9)	
H12A	0.8857	0.2776	0.6735	0.063*	
H12B	0.8531	0.2321	0.5898	0.063*	
H12C	0.8151	0.1401	0.7072	0.063*	
C13	0.3489 (3)	0.6528 (3)	0.8329 (2)	0.0271 (7)	
H13A	0.3865	0.7092	0.7648	0.033*	
C14	0.3850 (3)	0.6718 (3)	0.9109 (2)	0.0290 (7)	
H14A	0.4462	0.7401	0.8964	0.035*	
C15	0.3314 (3)	0.5907 (3)	1.0113 (2)	0.0284 (7)	
C16	0.2466 (3)	0.4899 (3)	1.0274 (2)	0.0268 (7)	
H16A	0.2111	0.4304	1.0945	0.032*	
C17	0.2136 (3)	0.4755 (3)	0.9458 (2)	0.0235 (6)	
C18	0.1223 (3)	0.3723 (3)	0.9571 (2)	0.0229 (6)	
C19	0.0696 (3)	0.2746 (3)	1.0491 (2)	0.0267 (6)	
H19A	0.0920	0.2722	1.1088	0.032*	
C20	−0.0157 (3)	0.1802 (3)	1.0546 (2)	0.0284 (7)	
C21	−0.0451 (3)	0.1869 (3)	0.9656 (3)	0.0309 (7)	
H21A	−0.1036	0.1243	0.9663	0.037*	
C22	0.0116 (3)	0.2859 (3)	0.8754 (2)	0.0291 (7)	
H22A	−0.0090	0.2892	0.8148	0.035*	
C23	0.3648 (4)	0.6126 (4)	1.0986 (3)	0.0380 (8)	
H23A	0.3052	0.5570	1.1637	0.057*	
H23B	0.3434	0.7046	1.0933	0.057*	
H23C	0.4675	0.5905	1.0955	0.057*	
C24	−0.0717 (4)	0.0744 (3)	1.1545 (3)	0.0384 (8)	
H24A	−0.1447	0.0271	1.1483	0.058*	
H24B	−0.1156	0.1133	1.2080	0.058*	
H24C	0.0085	0.0135	1.1724	0.058*	
Cl2	0.35132 (10)	0.10613 (9)	0.13262 (9)	0.0482 (3)	
O1S	0.3730 (3)	0.1709 (3)	0.3197 (2)	0.0526 (7)	
H1S	0.3480	0.1380	0.2767	0.063*	
O1W	0.3211 (3)	0.0038 (3)	0.9589 (2)	0.0505 (7)	
H1W	0.2964	0.0315	1.0088	0.061*	
H2W	0.4102	−0.0247	0.9268	0.061*	
C1S	0.2544 (5)	0.2599 (4)	0.3334 (3)	0.0511 (10)	
H1SA	0.2162	0.2901	0.2726	0.061*	
H1SB	0.2877	0.3371	0.3391	0.061*	
C2S	0.1359 (5)	0.2007 (6)	0.4266 (3)	0.0722 (15)	
H2SA	0.0615	0.2685	0.4382	0.108*	
H2SB	0.1756	0.1620	0.4859	0.108*	
H2SC	0.0927	0.1326	0.4170	0.108*	

### Atomic displacement parameters (Å^2^)

**Table:**

	*U*^11^	*U*^22^	*U*^33^	*U*^12^	*U*^13^	*U*^23^
V1	0.0275 (3)	0.0332 (3)	0.0219 (3)	−0.0078 (2)	−0.0058 (2)	−0.0050 (2)
Cl1	0.0360 (5)	0.0488 (5)	0.0279 (4)	−0.0096 (4)	−0.0037 (3)	−0.0165 (4)
O1	0.0276 (11)	0.0311 (11)	0.0173 (10)	−0.0014 (9)	0.0052 (8)	−0.0089 (9)
N1	0.0318 (14)	0.0295 (14)	0.0196 (12)	−0.0053 (11)	−0.0031 (11)	−0.0102 (11)
N2	0.0328 (14)	0.0264 (13)	0.0188 (12)	−0.0083 (11)	−0.0039 (11)	−0.0078 (10)
N4	0.0222 (13)	0.0311 (14)	0.0208 (12)	−0.0054 (10)	−0.0019 (10)	−0.0086 (11)
N3	0.0203 (12)	0.0307 (13)	0.0196 (12)	−0.0037 (10)	−0.0018 (10)	−0.0088 (10)
C1	0.0371 (18)	0.0299 (17)	0.0264 (16)	−0.0033 (14)	−0.0077 (14)	−0.0094 (13)
C2	0.043 (2)	0.0282 (16)	0.0235 (16)	−0.0093 (14)	−0.0060 (14)	−0.0045 (13)
C3	0.043 (2)	0.0338 (17)	0.0197 (15)	−0.0132 (15)	−0.0029 (14)	−0.0100 (13)
C4	0.0326 (17)	0.0341 (17)	0.0258 (16)	−0.0071 (14)	−0.0014 (13)	−0.0153 (14)
C5	0.0341 (17)	0.0289 (16)	0.0192 (14)	−0.0075 (13)	−0.0055 (12)	−0.0107 (12)
C6	0.0307 (16)	0.0304 (16)	0.0188 (14)	−0.0069 (13)	−0.0023 (12)	−0.0120 (12)
C7	0.0307 (17)	0.0334 (17)	0.0248 (16)	−0.0082 (13)	0.0006 (13)	−0.0122 (13)
C8	0.0311 (17)	0.0384 (18)	0.0293 (16)	−0.0005 (14)	−0.0070 (13)	−0.0200 (14)
C9	0.0348 (18)	0.0301 (16)	0.0271 (16)	−0.0024 (14)	−0.0057 (14)	−0.0125 (13)
C10	0.0310 (17)	0.0276 (16)	0.0241 (15)	−0.0063 (13)	−0.0021 (13)	−0.0099 (13)
C11	0.051 (2)	0.0370 (19)	0.0270 (17)	−0.0181 (17)	0.0017 (16)	−0.0083 (15)
C12	0.0327 (19)	0.048 (2)	0.045 (2)	0.0002 (16)	−0.0086 (16)	−0.0181 (18)
C13	0.0263 (16)	0.0268 (15)	0.0248 (15)	−0.0061 (12)	−0.0012 (12)	−0.0076 (13)
C14	0.0266 (16)	0.0314 (17)	0.0303 (16)	−0.0056 (13)	−0.0029 (13)	−0.0142 (14)
C15	0.0262 (16)	0.0322 (17)	0.0306 (17)	0.0024 (13)	−0.0085 (13)	−0.0156 (14)
C16	0.0269 (16)	0.0305 (16)	0.0221 (15)	0.0023 (13)	−0.0050 (12)	−0.0105 (13)
C17	0.0211 (15)	0.0262 (15)	0.0224 (15)	0.0007 (12)	−0.0041 (12)	−0.0094 (12)
C18	0.0211 (14)	0.0243 (15)	0.0225 (14)	0.0020 (11)	−0.0043 (12)	−0.0093 (12)
C19	0.0285 (16)	0.0271 (15)	0.0221 (15)	−0.0012 (12)	−0.0040 (12)	−0.0077 (12)
C20	0.0245 (15)	0.0247 (15)	0.0284 (16)	−0.0006 (12)	0.0021 (13)	−0.0078 (13)
C21	0.0254 (16)	0.0291 (16)	0.0347 (18)	−0.0055 (13)	−0.0035 (13)	−0.0089 (14)
C22	0.0266 (16)	0.0340 (17)	0.0300 (17)	−0.0069 (13)	−0.0062 (13)	−0.0135 (14)
C23	0.045 (2)	0.043 (2)	0.0341 (19)	−0.0044 (16)	−0.0155 (16)	−0.0176 (16)
C24	0.041 (2)	0.0316 (18)	0.0310 (18)	−0.0087 (15)	0.0018 (15)	−0.0037 (15)
Cl2	0.0400 (5)	0.0330 (5)	0.0766 (7)	0.0011 (4)	−0.0211 (5)	−0.0208 (5)
O1S	0.0405 (15)	0.0630 (18)	0.0399 (15)	−0.0082 (13)	−0.0101 (12)	0.0009 (13)
O1W	0.0354 (14)	0.0557 (17)	0.0618 (18)	−0.0113 (12)	−0.0108 (13)	−0.0195 (14)
C1S	0.055 (3)	0.048 (2)	0.048 (2)	−0.004 (2)	−0.022 (2)	−0.0080 (19)
C2S	0.058 (3)	0.102 (4)	0.040 (2)	0.012 (3)	−0.010 (2)	−0.012 (3)

### Geometric parameters (Å, º)

**Table:**

V1—O1	1.643 (2)	C12—H12B	0.9800
V1—N4	2.112 (3)	C12—H12C	0.9800
V1—N1	2.122 (3)	C13—C14	1.377 (5)
V1—N3	2.126 (3)	C13—H13A	0.9500
V1—N2	2.278 (3)	C14—C15	1.392 (5)
V1—Cl1	2.3259 (10)	C14—H14A	0.9500
N1—C1	1.349 (4)	C15—C16	1.388 (5)
N1—C5	1.355 (4)	C15—C23	1.508 (4)
N2—C10	1.343 (4)	C16—C17	1.387 (4)
N2—C6	1.351 (4)	C16—H16A	0.9500
N4—C22	1.339 (4)	C17—C18	1.474 (4)
N4—C18	1.362 (4)	C18—C19	1.384 (4)
N3—C13	1.334 (4)	C19—C20	1.387 (4)
N3—C17	1.360 (4)	C19—H19A	0.9500
C1—C2	1.381 (5)	C20—C21	1.385 (5)
C1—H1B	0.9500	C20—C24	1.499 (4)
C2—C3	1.392 (5)	C21—C22	1.384 (5)
C2—H2A	0.9500	C21—H21A	0.9500
C3—C4	1.393 (5)	C22—H22A	0.9500
C3—C11	1.509 (4)	C23—H23A	0.9800
C4—C5	1.385 (4)	C23—H23B	0.9800
C4—H4A	0.9500	C23—H23C	0.9800
C5—C6	1.480 (4)	C24—H24A	0.9800
C6—C7	1.392 (5)	C24—H24B	0.9800
C7—C8	1.384 (5)	C24—H24C	0.9800
C7—H7A	0.9500	O1S—C1S	1.408 (5)
C8—C9	1.388 (5)	O1S—H1S	0.9310
C8—C12	1.508 (5)	O1W—H1W	0.8444
C9—C10	1.382 (5)	O1W—H2W	0.9188
C9—H9A	0.9500	C1S—C2S	1.493 (6)
C10—H10A	0.9500	C1S—H1SA	0.9900
C11—H11A	0.9800	C1S—H1SB	0.9900
C11—H11B	0.9800	C2S—H2SA	0.9800
C11—H11C	0.9800	C2S—H2SB	0.9800
C12—H12A	0.9800	C2S—H2SC	0.9800
			
O1—V1—N4	102.71 (10)	C8—C12—H12A	109.5
O1—V1—N1	94.54 (11)	C8—C12—H12B	109.5
N4—V1—N1	160.77 (11)	H12A—C12—H12B	109.5
O1—V1—N3	94.03 (11)	C8—C12—H12C	109.5
N4—V1—N3	77.27 (10)	H12A—C12—H12C	109.5
N1—V1—N3	93.27 (10)	H12B—C12—H12C	109.5
O1—V1—N2	165.96 (10)	N3—C13—C14	122.8 (3)
N4—V1—N2	88.49 (10)	N3—C13—H13A	118.6
N1—V1—N2	73.27 (10)	C14—C13—H13A	118.6
N3—V1—N2	80.04 (9)	C13—C14—C15	119.8 (3)
O1—V1—Cl1	98.86 (8)	C13—C14—H14A	120.1
N4—V1—Cl1	92.74 (8)	C15—C14—H14A	120.1
N1—V1—Cl1	92.94 (7)	C16—C15—C14	117.4 (3)
N3—V1—Cl1	165.19 (8)	C16—C15—C23	121.8 (3)
N2—V1—Cl1	88.91 (7)	C14—C15—C23	120.8 (3)
C1—N1—C5	118.0 (3)	C17—C16—C15	120.3 (3)
C1—N1—V1	121.3 (2)	C17—C16—H16A	119.8
C5—N1—V1	120.6 (2)	C15—C16—H16A	119.8
C10—N2—C6	117.4 (3)	N3—C17—C16	121.2 (3)
C10—N2—V1	126.8 (2)	N3—C17—C18	115.4 (3)
C6—N2—V1	115.8 (2)	C16—C17—C18	123.4 (3)
C22—N4—C18	118.1 (3)	N4—C18—C19	121.2 (3)
C22—N4—V1	125.9 (2)	N4—C18—C17	115.5 (3)
C18—N4—V1	115.9 (2)	C19—C18—C17	123.3 (3)
C13—N3—C17	118.5 (3)	C18—C19—C20	120.5 (3)
C13—N3—V1	125.8 (2)	C18—C19—H19A	119.8
C17—N3—V1	115.40 (19)	C20—C19—H19A	119.8
N1—C1—C2	123.4 (3)	C21—C20—C19	117.9 (3)
N1—C1—H1B	118.3	C21—C20—C24	122.0 (3)
C2—C1—H1B	118.3	C19—C20—C24	120.1 (3)
C1—C2—C3	118.8 (3)	C22—C21—C20	119.3 (3)
C1—C2—H2A	120.6	C22—C21—H21A	120.4
C3—C2—H2A	120.6	C20—C21—H21A	120.4
C2—C3—C4	117.9 (3)	N4—C22—C21	123.0 (3)
C2—C3—C11	121.2 (3)	N4—C22—H22A	118.5
C4—C3—C11	121.0 (3)	C21—C22—H22A	118.5
C5—C4—C3	120.5 (3)	C15—C23—H23A	109.5
C5—C4—H4A	119.7	C15—C23—H23B	109.5
C3—C4—H4A	119.7	H23A—C23—H23B	109.5
N1—C5—C4	121.3 (3)	C15—C23—H23C	109.5
N1—C5—C6	115.5 (3)	H23A—C23—H23C	109.5
C4—C5—C6	123.2 (3)	H23B—C23—H23C	109.5
N2—C6—C7	122.1 (3)	C20—C24—H24A	109.5
N2—C6—C5	114.7 (3)	C20—C24—H24B	109.5
C7—C6—C5	123.2 (3)	H24A—C24—H24B	109.5
C8—C7—C6	120.1 (3)	C20—C24—H24C	109.5
C8—C7—H7A	119.9	H24A—C24—H24C	109.5
C6—C7—H7A	119.9	H24B—C24—H24C	109.5
C7—C8—C9	117.6 (3)	C1S—O1S—H1S	99.6
C7—C8—C12	121.7 (3)	H1W—O1W—H2W	130.3
C9—C8—C12	120.8 (3)	O1S—C1S—C2S	112.2 (4)
C10—C9—C8	119.4 (3)	O1S—C1S—H1SA	109.2
C10—C9—H9A	120.3	C2S—C1S—H1SA	109.2
C8—C9—H9A	120.3	O1S—C1S—H1SB	109.2
N2—C10—C9	123.4 (3)	C2S—C1S—H1SB	109.2
N2—C10—H10A	118.3	H1SA—C1S—H1SB	107.9
C9—C10—H10A	118.3	C1S—C2S—H2SA	109.5
C3—C11—H11A	109.5	C1S—C2S—H2SB	109.5
C3—C11—H11B	109.5	H2SA—C2S—H2SB	109.5
H11A—C11—H11B	109.5	C1S—C2S—H2SC	109.5
C3—C11—H11C	109.5	H2SA—C2S—H2SC	109.5
H11A—C11—H11C	109.5	H2SB—C2S—H2SC	109.5
H11B—C11—H11C	109.5		
			
O1—V1—N1—C1	−8.2 (3)	V1—N1—C5—C6	−2.2 (3)
N4—V1—N1—C1	−162.1 (3)	C3—C4—C5—N1	1.1 (5)
N3—V1—N1—C1	−102.5 (2)	C3—C4—C5—C6	−178.7 (3)
N2—V1—N1—C1	178.9 (3)	C10—N2—C6—C7	−0.9 (4)
Cl1—V1—N1—C1	91.0 (2)	V1—N2—C6—C7	175.3 (2)
O1—V1—N1—C5	173.1 (2)	C10—N2—C6—C5	−180.0 (3)
N4—V1—N1—C5	19.2 (4)	V1—N2—C6—C5	−3.8 (3)
N3—V1—N1—C5	78.8 (2)	N1—C5—C6—N2	4.0 (4)
N2—V1—N1—C5	0.2 (2)	C4—C5—C6—N2	−176.2 (3)
Cl1—V1—N1—C5	−87.8 (2)	N1—C5—C6—C7	−175.2 (3)
O1—V1—N2—C10	147.3 (4)	C4—C5—C6—C7	4.7 (5)
N4—V1—N2—C10	4.0 (3)	N2—C6—C7—C8	0.5 (5)
N1—V1—N2—C10	177.8 (3)	C5—C6—C7—C8	179.6 (3)
N3—V1—N2—C10	81.3 (3)	C6—C7—C8—C9	0.0 (5)
Cl1—V1—N2—C10	−88.8 (2)	C6—C7—C8—C12	−179.9 (3)
O1—V1—N2—C6	−28.5 (5)	C7—C8—C9—C10	−0.1 (5)
N4—V1—N2—C6	−171.8 (2)	C12—C8—C9—C10	179.8 (3)
N1—V1—N2—C6	2.1 (2)	C6—N2—C10—C9	0.7 (4)
N3—V1—N2—C6	−94.4 (2)	V1—N2—C10—C9	−175.0 (2)
Cl1—V1—N2—C6	95.5 (2)	C8—C9—C10—N2	−0.3 (5)
O1—V1—N4—C22	90.6 (3)	C17—N3—C13—C14	−2.0 (5)
N1—V1—N4—C22	−116.2 (3)	V1—N3—C13—C14	171.4 (2)
N3—V1—N4—C22	−178.1 (3)	N3—C13—C14—C15	0.0 (5)
N2—V1—N4—C22	−98.0 (3)	C13—C14—C15—C16	2.2 (5)
Cl1—V1—N4—C22	−9.1 (3)	C13—C14—C15—C23	−177.7 (3)
O1—V1—N4—C18	−86.5 (2)	C14—C15—C16—C17	−2.3 (5)
N1—V1—N4—C18	66.7 (4)	C23—C15—C16—C17	177.6 (3)
N3—V1—N4—C18	4.8 (2)	C13—N3—C17—C16	1.8 (4)
N2—V1—N4—C18	84.9 (2)	V1—N3—C17—C16	−172.3 (2)
Cl1—V1—N4—C18	173.8 (2)	C13—N3—C17—C18	−178.8 (3)
O1—V1—N3—C13	−77.9 (3)	V1—N3—C17—C18	7.1 (3)
N4—V1—N3—C13	180.0 (3)	C15—C16—C17—N3	0.4 (5)
N1—V1—N3—C13	16.9 (3)	C15—C16—C17—C18	−179.0 (3)
N2—V1—N3—C13	89.3 (3)	C22—N4—C18—C19	1.1 (4)
Cl1—V1—N3—C13	131.5 (3)	V1—N4—C18—C19	178.4 (2)
O1—V1—N3—C17	95.7 (2)	C22—N4—C18—C17	−179.9 (3)
N4—V1—N3—C17	−6.5 (2)	V1—N4—C18—C17	−2.6 (3)
N1—V1—N3—C17	−169.5 (2)	N3—C17—C18—N4	−3.0 (4)
N2—V1—N3—C17	−97.2 (2)	C16—C17—C18—N4	176.4 (3)
Cl1—V1—N3—C17	−54.9 (4)	N3—C17—C18—C19	175.9 (3)
C5—N1—C1—C2	−0.4 (5)	C16—C17—C18—C19	−4.7 (5)
V1—N1—C1—C2	−179.1 (2)	N4—C18—C19—C20	−1.1 (5)
N1—C1—C2—C3	1.2 (5)	C17—C18—C19—C20	−179.9 (3)
C1—C2—C3—C4	−0.9 (5)	C18—C19—C20—C21	0.3 (5)
C1—C2—C3—C11	179.9 (3)	C18—C19—C20—C24	179.7 (3)
C2—C3—C4—C5	−0.3 (5)	C19—C20—C21—C22	0.4 (5)
C11—C3—C4—C5	178.9 (3)	C24—C20—C21—C22	−179.1 (3)
C1—N1—C5—C4	−0.8 (4)	C18—N4—C22—C21	−0.4 (5)
V1—N1—C5—C4	177.9 (2)	V1—N4—C22—C21	−177.5 (2)
C1—N1—C5—C6	179.0 (3)	C20—C21—C22—N4	−0.3 (5)

### Hydrogen-bond geometry (Å, º)

**Table:**

*D*—H···*A*	*D*—H	H···*A*	*D*···*A*	*D*—H···*A*
O1*S*—H1*S*···Cl2	0.93	2.24	3.128 (3)	159
O1*W*—H1*W*···Cl2^i^	0.84	2.45	3.221 (3)	152
O1*W*—H2*W*···Cl2^ii^	0.92	2.37	3.284 (3)	171
C1—H1*B*···O1	0.95	2.51	2.999 (4)	112
C2—H2*A*···O1*S*^iii^	0.95	2.41	3.301 (4)	155
C4—H4*A*···Cl1^iv^	0.95	2.76	3.670 (4)	160
C7—H7*A*···Cl1^iv^	0.95	2.77	3.626 (3)	151
C9—H9*A*···Cl2^ii^	0.95	2.63	3.561 (4)	165
C13—H13*A*···O1*S*^iv^	0.95	2.59	3.284 (4)	130
C14—H14*A*···Cl2^iv^	0.95	2.62	3.558 (3)	170
C19—H19*A*···O1^v^	0.95	2.52	3.229 (4)	132
C21—H21*A*···O1*W*^vi^	0.95	2.45	3.315 (5)	151
C22—H22*A*···Cl1	0.95	2.64	3.262 (3)	123

Symmetry codes: (i) *x*, *y*, *z*+1; (ii) −*x*+1, −*y*, −*z*+1; (iii) *x*, *y*+1, *z*; (iv) −*x*+1, −*y*+1, −*z*+1; (v) −*x*, −*y*+1, −*z*+2; (vi) −*x*, −*y*, −*z*+2.
